# How Good Are We at Detecting a Phishing Attack? Investigating the Evolving Phishing Attack Email and Why It Continues to Successfully Deceive Society

**DOI:** 10.1007/s42979-022-01069-1

**Published:** 2022-02-23

**Authors:** Fiona Carroll, John Ayooluwa Adejobi, Reza Montasari

**Affiliations:** 1 Cardiff Metropolitan University Llandaff Campus, Western Avenue, Cardiff, CF5 2YB UK; 2 Hillary Rodham Clinton School of Law, Swansea University, Singleton Park, Swansea, Wales SA2 8PP UK

**Keywords:** Phishing email attack, COVID-19, Cyber security, Human factors

## Abstract

Phishing attacks are on the increase. The fact that our ways of living, studying and working have drastically changed as a result of the COVID pandemic (i.e., almost everything being done online) has created many new cyber security concerns. In particular, with the move to remote working, the number of phishing emails threatening employees has increased. The 2020 Phishing Attack Landscape Report (Greathorn: 2020 Phishing attack landscape. https://info.greathorn.com/report-2020-phishing-attack-landscape/, 2020) highlights a sharp increase in the frequency of attempted phishing attacks. In this paper, we are interested in how the phishing email attack has evolved to this very threatening state. In detail, we explore the current phishing attack characteristics especially the growing challenges that have emerged as a result of the COVID-19 pandemic. The paper documents a study that presented test participants with five different categories of emails (including phishing and non phishing) . The findings from the study show that participants, generally, found it difficult to detect modern phishing email attacks. Saying that, participants were alert to the spelling mistakes of the older phishing email attacks, sensitive information being requested from them and any slight change to what they were normally used to from an email. Moreover, we have found that people were not confident, worried and often dissatisfied with the current technologies available to protect them against phishing emails. In terms of trust, these feelings alerted us to the increasing severity of the phishing attack situation and just how vulnerable society has become/ still is.

## Introduction

During the last year and a half of lockdown and COVID, emails have become far more important than they ever were before. For many they have become the lifeline for sustaining communication and contact with friends, family and work colleagues. In fact, email has become of ‘critical importance as a communication channel for both business and personal matters’s [21, p.1]. Unfortunately along with the genuine email communications comes phishing emails. As Williams and Joinson [51, p.1] note ‘Phishing e-mails are fraudulent e-mails used to gain access to sensitive information or secure computer systems... They persuade users to click on malicious links, download attachments or provide sensitive information, such as usernames or passwords’. There is no doubt that phishing email attacks have been around for several decades but they somehow they have evolved to be a major problem today in that they constitute a severe threat in the cyber world [3]. As Basit et al. [7, p.1] highlights ‘In recent times, a phishing attack has become one of the most prominent attacks faced by internet users, governments, and service-providing organizations’. One of the main reasons for this is that when the premise of a phishing email aligns with a user’s work context, it is much more challenging for users to detect a phish [41, p.1]. Artificial Intelligence (AI) techniques such as machine learning, deep learning, hybrid learning, and scenario-based techniques are all being urgently explored to try to solve this phishing email pandemic.

However, despite the ‘availability of myriads anti-phishing systems, phishing continues unabated due to inadequate detection of a zero-day attack, superfluous computational overhead and high false rates’ [30, p.1]. Moreover, in their research, Wash [50] found that while technical protections against phishing reduce the number of phishing emails received, they are not perfect. This could be because individual’s phishing susceptibility may be shaped by recent phishing encounters and, more importantly, that the effect of new experience on susceptibility will be heterogeneous among users [9, p.1]. To better understand the cognitive process that end users can use to identify phishing messages, Wash [50] interviewed number of IT experts about where they successfully identified emails as phishing in their own inboxes. The problem is ‘the variety of phishing attacks is very broad, and usage of novel, more sophisticated methods complicate its automated filtering’ [33, p.1]. To complicate things further, the use of social network sites (SNSs) entice users to click on malicious links masquerading as fake news, controversial videos and other opportunities thought to be attractive or beneficial to the victim [14, p.1]. Therefore as Chiew [10, p.1] points out there ‘is a need for a review of the past and current phishing approaches’. This paper will give a snapshot of the current cyber security landscape with a focus on the evolving phishing email attack and COVID-19. It will document the findings from a study which investigated people’s perceptions of the phishing email attack both current and past. The paper will conclude with a discussion on the main points of interest from the study and literature, with a reflection on where we go from here.

## Cyber Security Landscape 2020/2021

Cyber security is about reducing the risk of a cyber attack; it examines the protection of computer networks and systems from data disclosure, software and/ or hardware damage etc. COVID-19, however, has had a huge impact on cyber security. During the pandemic several industries have been targeted by attackers for malicious purposes. ‘While all industries have been concentrated, experts have acknowledged details focusing on healthcare, education, manufacturing, media, advertising, and hospitality organizations in certain campaigns’ [28, p.4]. Indeed, the cyber security of organizations is continually susceptible to attacks and COVID-19 has shown that these challenges keep evolving. ‘One of the most problematical components in reference to cyber security will be the frequently evolving nature of security perils’ [28, p.4]. Cyber defense suffers due to the anonymous form of cyber attacks as in most cases, there is no real time warning. ‘The anonymity or ‘attribution’ problem is serious enough that it increases the odds that damaging cyber attacks on national critical infrastructures will take place in the absence of any traditional, real-world warning, during times of nominal peace’ [16, p.11]. Looking into 2021 and beyond, cyber criminals are rife and phishing email attacks are a key player in this digital threat landscape. Particularly, as they continue to successfully to evade both technical and human defenses. As Shackleton [36, p.1.] notes ‘organisations should expect phishing to remain one of the main threat vectors that hackers use to deliver both ransomware and business email compromise (BEC) attacks in 2021’.

### The Evolution of the Phishing Attack and COVID-19

In today’s world, the digital is fully immersed into our society. This in itself brings huge challenges as people have little option, particularly as we have seen during COVID-19, other than to embrace this way of living and all its new technologies. It cannot be denied that the digital can bring huge benefits; it has enabled us to work, study, socialize and live during lockdowns. However, it also means that we must live with the possibility of being scammed or in hope that solutions will come in play to totally protect us. As Lallie et al. [27] noted the cyber crime occurrence from the pandemic poses serious threats to the safety of the global economy. And phishing email attacks are one of the top cyber crime occurrences and top cyber security threats. Phishing attacks have been the most common crime from 2020, with phishing incidents nearly doubled in regularity [13]. Business Email Compromise attacks are the most common and amount to huge losses. These phishing attacks come in the form of a request, urgent, important, seeking attention and often requiring some form of payment [44]. According to research some industries are more targeted than others, for example, public administration services had the most breaches from social engineering, followed by other professional services [49]. In addition, during the first quarter of 2021, there were a lot of popular brands impersonated e.g. Google, LinkedIn, Amazon etc. [35]. These are some of the platforms that people rely on for their day-to-day activities particularly, as many restrictions were in place due to the pandemic. Cyber security experts identified in 2021 that attackers bamboozle victims with the ‘unsubscribe’ caption in their mails [1]; they are clever and strategic in their execution. These captions in the mail leads to further spam, it is being used to confirm the validity of the mail as victims click ‘unsubscribe’ [1].

As technology continues to evolve, attackers are also becoming more creative in how they scam people and more sophisticated in the techniques they use to persuade their victims. As Vayansky et al. [48, p.3] note ‘Phishers have become more skilled at forging websites to appear identical to the expected location, even including logos and graphics in the phishing emails to make them more convincing’. There are many tricks that phishers use to manipulate their victims and to get sensitive data from them. Some common properties of phishing attacks in websites are found in logos, suspicious URLs, https, images etc. [4, p.4]. However, these 2021 phishing attack characteristics are becoming harder to pinpoint. Along with this, is the frequency of attacks and how widespread they have become. According to Bartoli et al. [6] ‘Recent phishing campaigns are increasingly targeted to specific, small population of users and last for increasingly shorter life spans’. Therefore, there is less time for detecting Zero day phishing attacks. In addition, phishing ‘affects people globally and is conducted internationally, making it difficult to track and prosecute the criminals behind it’ [48, p.4].

One of the reasons for this recent difficulty in detecting phishing emails, has been a significant use of COVID-19 themes for phishing and online fraud. Malicious attackers have taken COVID-19 as an opportunity to launch attacks for financial gains and to promote their evil intents... and many people are falling prey to phishing attacks [25]. As Interpol [20] reported, there were 907,000 spam COVID-19-related messages and 48,000 malicious URLs detected between January and April, 2020. It was found that the growth in anxiety and fear due to the pandemic increased the success rate of cyber-attacks and interestingly healthcare organizations were one of the main victims of cyber-attacks during the pandemic [32]. Research [17] suggests clearly that COVID-19 restrictions generated the increase in phishing. Moreover, the findings from the UK Cyber Security Breaches Survey 2021 [23] mirrors this when it highlights that for businesses there has been a rise in phishing attacks (from 72 to 83%) from 2017 to 2021. Research by Symantec [45] shows that throughout 2020, 1 in every 4200 emails was a phishing email. Without a doubt, the phishing attack email evolved further during the COVID-19 crisis. The pandemic (i.e., home working, online shopping, etc.) provided many opportunities to develop new tactics and techniques to prey on people’s emotions of fear, anxiety and their need for information around the pandemic. And this was the case the world over, for example, in a recent *Phishing Insights 2021* study, 90% of respondents in Israel reported an increase in phishing whilst Austria had a 88% increase and the UK a 74% increase in phishing attacks [39].

## Phishing Attacks and the Human Factor

The success of more recent phishing attacks depends on how convincing and often how familiar or relatable an email scenario is. As Parsons et al. [31, p.31] highlight: ‘social engineers will often use a contrived situation or personal persuasion to increase the chance that their request will be successful’. It is about how successful the email is at engaging the victim and then its ability to trigger a response. Indeed, attackers frequently manipulate situations, forcing victims to fall into error by influencing them to make bad decisions. Benenson et al. [8, p.1] note ‘Attack strategies include controlling and operating fake or compromised social media accounts, artificially manipulating the reputation of online entities, spreading false information, and manipulating users via psychological principles of influence into performing behaviors that are counter to their best interests and benefit the attackers’. Phishing attackers think nothing of using intimidation tactics; they will send well crafted emails to entrap and exploit their victim’s fears, interests and/ or curiosity. It is quite usual for them to ‘drop names of important people within the organization and the listener will find that they often make small mistakes about details or information.’ [31, p.38]. As a result, human error has been identified to have a high impact on the success of phishing attacks.

In reality, everyone can make mistakes no matter how well trained they are. Phishing attackers prey on human weakness and use it to lure their victims into a false sense of security. A study by Stanford University Professor Jeff Hancock and security firm Tessian [47] highlights that one in four employees (25%) said they have clicked on a phishing email at work, they found that men were twice as likely as women to fall for phishing scams and older employees were the least susceptible to phishing scams. In addition, as attackers engage with victims using various different mediums, these attacks have become more versatile in how they trick victims into giving up sensitive personal information. As Parsons et al. [31, p.1] emphasize ‘human factors play a significant role in computer security; factors such as individual difference, cognitive abilities and personality traits can impact on behavior. Information security behaviors are also greatly influenced by an individual’s perception of risk.’ It is from this perspective that the importance of the human factors and its role in the phishing attack experience comes to the surface [19]. As discussed previously, researchers show that ‘fear of COVID-19 influences the success of COVID-19 specific themed phishing scams, while anxiety, stress, and risk-taking influences the success of both the COVID-19 themed and common phishing scams’ [2, p.1].

Saying that research does also show that well-informed and trained users are the best defense against phishing. In detail, Singh et al. [38] studied people’s success on the detection of phishing emails and found that participants receiving higher frequency of phishing emails had a higher hit rate compared to participants encountering lower frequency levels during training. Another study [22] found that participants who received mindfulness training were better able to avoid the phishing attack. Sheng et al [37] found that the participants who played an online game (that teaches users good habits to help them avoid phishing attacks) were better able to identify fraudulent web sites compared to the participants in other conditions. In terms of anti phishing training, Sumner et al. [43] examined factors impacting the effectiveness of anti-phishing training and found that that the participant’s average accuracy in detecting phishing URLs increased 8% and their confidence in their answer choices increased 6% from pre-training to post-training surveys. Interestingly, they also found that participants with the Influence personality trait had the lowest susceptibility while both Dominant and Steadiness personalities had the highest susceptibility before and after training respectively [43]. The important point here is that phishing can mean different things to different people. For example, 57% of IT professionals studied felt that a phishing attack was an email that falsely claimed to be from a legitimate organization, usually combined with a threat or request for information whilst 49% of respondents consider an email with a malicious link to be phishing [39]. Cultural factors have a significant impact on people’s understanding of phishing and this is important to be aware of when trying to improve the human’s efficiency in phishing email detection.

### The Victim

Personality, culture, gender, age, expertise, workload, stress and vigilance are all factors that researchers have considered when trying to work out why humans are susceptible to phishing attacks, and then how we can minimize or at least mitigate their damage [29]. Indeed, studies have found that the ‘so called phishing susceptibility (i.e., the likelihood of being phished) is closely correlated with the individuals’ personality traits’ [11, p.1]. As Frauenstein et al. [14, p.1] highlight ‘conscientious users were found to have a negative influence on heuristic processing and are thus less susceptible to phishing on SNSs... heuristic processing increases susceptibility to phishing’. As mentioned, cultural background can also influence the level of one’s security awareness with factors such as language, location playing a key role etc. As Kruger et al. [26, p.1] highlight ‘cultural factors such as mother tongue, area where you grew up, etc., do have an impact on security awareness levels’. And these culture affects then have an impact on privacy and trust attitudes, which indirectly affect one’s susceptibility to these cyberattacks [29].

Some research shows that gender also has a role to play on one’s susceptibility to the phishing attack. In earlier studies, females have often been found to be more trusting and vulnerable to phishing websites than males. As Alseadoon et al. [5, p.2] note ‘The reason for females’ vulnerability contributes to three factors: females have less knowledge about security procedures; Females are more susceptible than males; Females are more welling towards trust’. However, Montañez et al. [29] emphasizes that gender does not have a big impact on the susceptibility to social engineering cyberattacks. Moreover, phishing attack susceptibility can be strongly correlated to an individuals’ lack of knowledge, experience and/or education in cyber security. Often, it requires technical knowledge to fully comprehend the depth of the security risk. As Parsons et al. [31, p.16] state ‘Since an individual may view their actions on their personal computer to be under their control, threats may be seen as less risky... this means that individuals might be more likely to engage in risky behavior’. Attackers exploit these behaviors as well as the tendency to be too trusting especially towards certain reputable-looking websites designed to lure users to disclose sensitive data. As Alseadoon et al. [5, p.2] point out,'user’s habit of trust makes users examine fewer cues or deceived by deceptive cues’. They ultimately result in people losing total trust with online platforms. A study carried out with 155 participants shows ‘the consequences of phishing attacks go beyond financial loss, with many participants describing social ramifications such as embarrassment and reduced trust’ [24, p.1]. Indeed, ‘old people with higher education, higher awareness and higher exposure to social engineering cyberattacks are less susceptible to these attacks’ [29, p.1]. Saying that, things like, heavy workloads and stress can further impact the effect on people’s susceptibility and decision making around phishing attacks. Interestingly, Abroshan et al. [2] found that the attitude to risk-taking can predict users’ phishability. Yet, it is clear from the research that stress affects people’s decision-making [40].

### The Attacker

A measurement of how successful a phishing attack is can be bench-marked against how effective the phisher/attacker is in persuading and manipulating victims into conforming to their goals. In terms of the criminal character, a study by Gaia et al. [15, p.1] found evidence that ‘Grey Hatters oppose authority, Black Hatters score high on the thrill-seeking dimension and White Hatters, the good guys, tend to be Narcissists’. Phishing attackers are good at building up a false sense of trust, they are confidant at luring victims and influencing behavior. ‘Phishers are able to use similar methods to entice social network users to click on malicious links masquerading as fake news, controversial videos and other opportunities thought to be attractive or beneficial to the victim’ [14, p.1]. Human personality traits significantly contribute to the probability that an individual is susceptible to manipulation related to social engineering deception attacks and exploits [42]. The phisher can quickly detect these characteristics and social media has been identified as an avenue for supporting this detection. Many attackers have used such forms to manipulate and take original information out to context to promote misinformation and also to get sensitive data [52].

The way attackers influence their victims’ decision to believe false information is an art. The attacker creates a false relationship with the victim in order to increase the chance that the victim divulges private information to the attacker [12, p.5]. In general, people seem to be more susceptible to friends and/ or a familiar interface. Attackers know this and are very skillful in building this relationship and can be very deceptive in appearing as someone familiar. Moreover, people can be eager to comply with requests from platforms they like. The attackers are also aware of this and they use this method to get data (i.e., they bring up common topics or initiate discussions about common enemy, etc.) [12]. Phishing email attackers are not all the same but in different ways, they are all masters at influencing behavior by manipulating human emotion and trust. And often the amount of effort they put into an attack pays off. As Montañez et al. [29, p.1] highlights ‘Message quality and message appeal, which reflect attacker effort (e.g., using contextualization and personalization), have a significant impact on the attacker‘s success’.

## Study

The aim of this study is to give insight into individuals’ perception of the evolving phishing email attack. The authors of the paper were interested to investigate if people’s ability to detect phishing attacks has changed with every new evolution of the phishing email attack. They were also curious to understand the impact (if any) this has/could have on their person. Fifty-two participants completed this study. It took approximately ten minutes in duration and was approved by the Ethics Board of School of Technologies, Cardiff Met University.

**Fig. 1:**
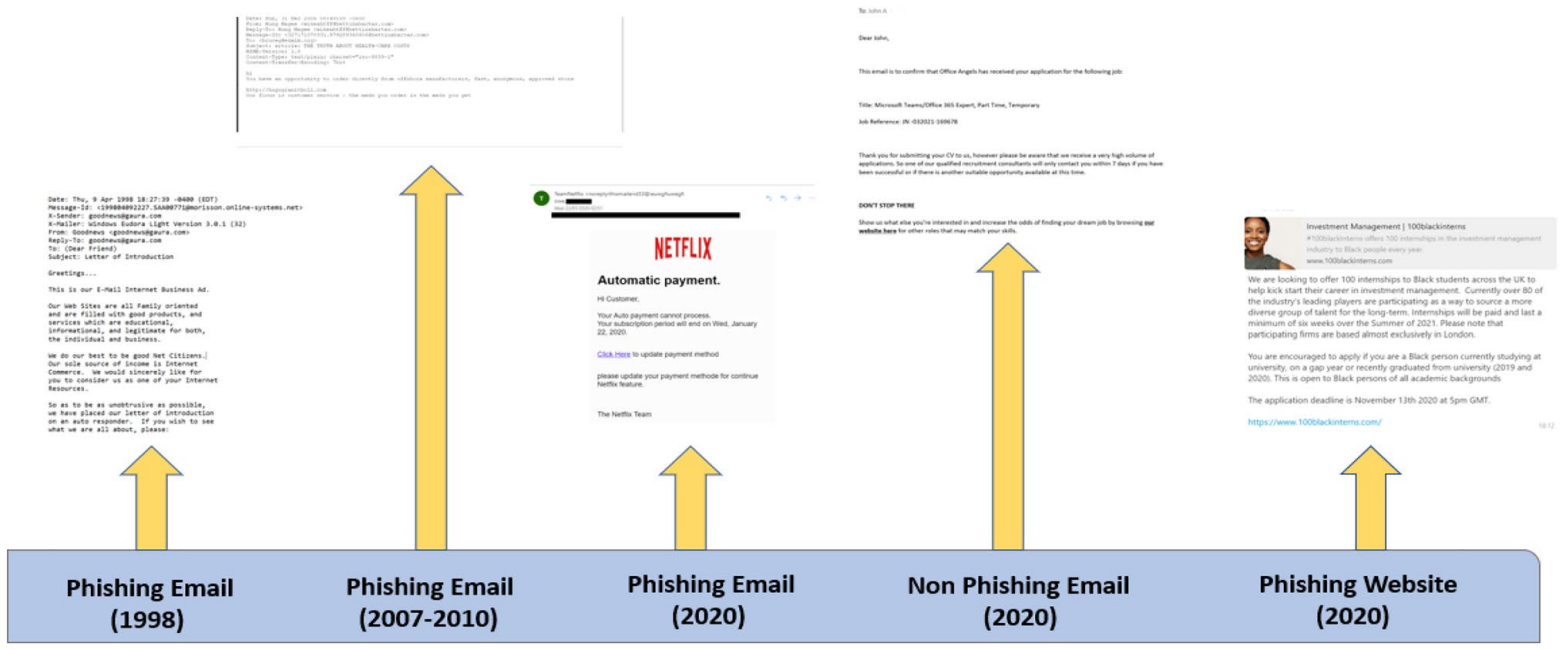
Five categories of phishing and non-phishing emails and website

### Research Design and Methodology

**Fig. 2:**
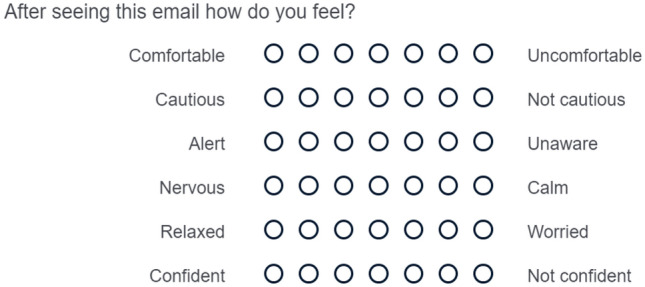
Semantic differential style question

A questionnaire was developed using Qualtrics survey software to investigate people’s perception around the detection of phishing email attacks. The study examined different ages (17–55 years old), educational groups (GCSE to Master’s degree), race and gender from across two countries (UK and Nigeria). This study was two-fold. The first part of the study consisted of a series of questions to probe participants online usage. These included some general questions such as: Do you have an email account?, Do you have a social media account?, How many hours do you think you spend online daily? Part 2 of the questionnaire randomly presented five different categories of emails to the participant. These included emails from category 1: phishing emails (1998); category 2: phishing emails (2007–2010); category 3: phishing emails (2020); category 4: an authentic non phishing email and finally, category 5: a phishing website (see Fig. 1). After each email, the participant was asked to complete a semantic differential style question and then to determine whether they felt the email was a phishing or non phishing email attack (see Fig. 2). A qualitative data analysis was undertaken to probe participant’s thinking and behavior around these different categories.

### Findings

**Fig. 3:**
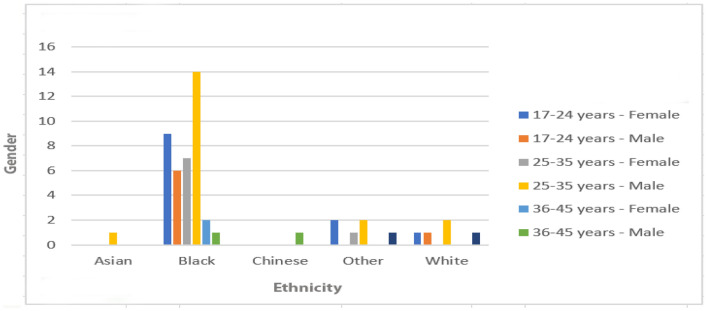
Demographic of test participants

**Fig. 4:**
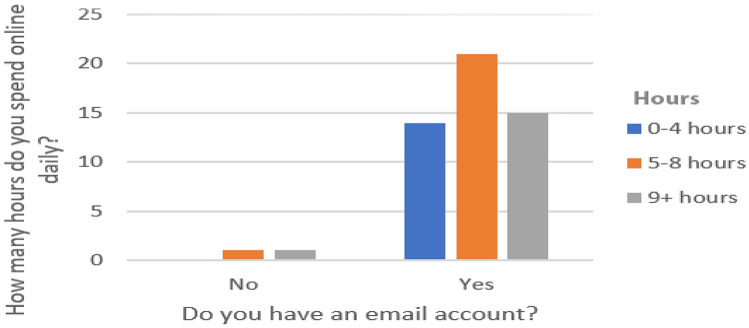
How many hours do test participants spend online daily?

From the 52 people who participated in the study, twenty-two were female and thirty were male (see Fig. 3 for a more detailed breakdown). The majority of these were well educated with 25 participants having a bachelors degree, five with a college degree, fourteen with a masters degree, seven with a high school degree and only one with no formal education. Moreover, fifty of the fifty-two test participants had an email account and fourteen of these spend 0–4 h online daily, twenty-one of these spend 5–8 h online daily and finally fifteen of these spend nine hours of more online daily (see Fig. 4).

It is clear from the findings that most participants (50%) could identify an older phishing email (category 1) by spellings and obvious grammar irregularities. As participants felt: ‘It does not have a normal email format’ (P.53) and ‘Because it just looks like a cyber-attack. I guess. It isn’t direct and also looks like a ‘code’(P.7). The findings show that participants became more alert and nervous as they came in contact with newer examples (category 2: 2007–2010). Thirty-one participants were unsure if it was a phishing email or not and stated their reasons:‘no major signs’ (P.20); ‘Looks weird’ (P.22); ‘Phishing emails usually contain a link’ (P.27); ‘I’m just not sure if it’s from a verified source’ (p.38) and ‘I don’t really know how phishing mails appear, but this feels weird’ (P.44). Interestingly, two participants did not believe it to be a phishing email. They supported their decisions with various reasons: ‘No claim for money’ (P.13) and ‘Because the content is just a phone number. They’ve got nothing’ (P.39).

In terms of the category 3 emails (2020), participants had many different experiences compared to those with the older examples. Some participants (20%) made the decision that it was not a phishing email. As the findings highlight: ‘It looks like a regular mail’ (P.8); ‘It’s from PayPal with a correct PayPal header and good email name I believe it’s true’ (P.26) and ‘It’s a reputable source’ (P.47). Other participants (14%) were unsure about the email: ‘It might be scam’ (P.10). Interestingly those who believed it was a phishing email had more leaning towards the email content. For example, Participant 14 believed it was a phishing email because of the layout. Other participants recognized that the ‘senders email address isn’t from PayPal’ (P.20) and ‘The senders address is not the official PayPal as the spelling is wrong’ (P.23). Many found it ‘Suspicious’ (P.28) and felt that ‘PayPal wouldn’t use an email address ending with @outlook.com. Also, PayPal seems misspelled ... ‘ (P23).

Most participants (46%) agreed that the category 4 email was not a phishing email as they did not see any irregularities. Some of the comments included: ‘Because it is well composed and addressed to someone’ (P.8). Many participants believed that it was an authentic email because of it’s formality and structure: ‘It is easy to understand and the information is properly communicated’ (P.15), ‘The email content looks like a response to a previous job application process’ (P.21). Several participants (22%) were unsure about the email considering it to be legit but still unsure: ‘It seems official but the link at the end of the email is rather suspicious’ (P.23) and ‘looks genuine but could also be a phishing mail’ (P.48).

Finally, participants were also presented with a phishing site example (category 5). Interestingly, many participants (31%) believed it was a legitimate site, attributing their choice to the presentation of the email: ‘looks genuine’(P.20), ‘It looks completely safe and legitimate it seems like a good scheme and idea and one that should be promoted’ (P.26). Other participants (29%), however, were unsure about the site (i.e., the link for the site gave them a feeling of uncertainty): ‘Need to visit it first, and see what they want’ (P.9).

**Fig. 5:**
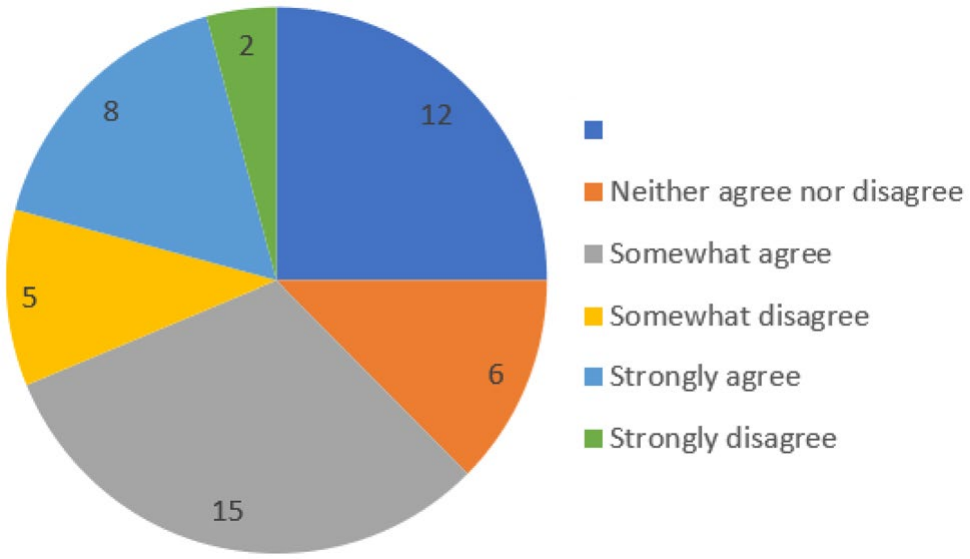
In your opinion do you think grammar, source, and content is enough to detect a phishing email attack?

### Discussion

Phishing is a prolific type of attack that has evolved steadily over the years and the findings have effectively highlighted this. It is clear from the data collected that participants were more confidant in deciding that the older email attacks were actual attacks compared to the more recent examples. Interestingly, thirty-one participants agreed to the possibility of phishing attacks being contextual. Moreover, twenty-three participants felt that social media has a huge impact on the success of the phishing attack whilst twenty-one participants firmly believed that current trends pose a difficulty for them to detect phishing attack. Worryingly, several participants (twenty-three participants) still believed that grammar, source, and content is enough to detect a phishing email attack (see Fig. 5). In fact, the authors found it very surprising to see that there was still such a strong emphasis on the appearance of the emails as a means to detect or not detect the attack. In terms of age, it was interesting to discover that the younger participants in the study found the older phishing emails (1998) to be strange (i.e., words such as weird were used frequently to describe their experience of the email). Overall, the findings clearly highlight the power of the evolving phishing email attack to continue to deceive society. And, it was very revealing to see that participants still felt that they needed: ‘Education on phishing’ (P.12); ‘Awareness’ (P.21); ‘Email platforms need to do more to protect their users by detecting these dubious emails and warning users about them’ (P.44) and ‘More awareness, more education on these attacks’ (P.48).

## Conclusion

The concept of phishing has been around for as long as email and with age comes maturity showing the phishing attack to be one of the most enduring cyber attacks out there. Over the last thirty plus years, the phishing attack has improved in its ability to appear legitimate and deceive the average person and/or business. In fact, it has grown into one of the most dangerous of all the cyber attacks. As we have seen in this study, many participants became alert and nervous as they came in contact with newer examples of the phishing attack emails. For many, this nervous disposition emerged as they could not decipher if the email was threat or not. In the physical world, we would experience acute stress as an immediate reaction to a threatening situation. Why should the virtual world be any different? The authors feel that the key lies in how we can support the end-user to ensure that they can fully comprehend the threat and be in control of the decision they make around this threat.

As technology advances, this nervousness is only likely to increase as the number and severity of the phishing email attack evolves. Tahir [46, p.1] highlights ‘the enduring success of phishing is simply a result of our human tendency to be tempted to perform an action, especially when we trust the source’. Trust is another key player in these experiences; however, it can be two sided. One of the biggest impacts of the COVID-19 pandemic was the trust that people put into technology to maintain a ‘normality’ ( i.e., a work, school and socializing normality). Unfortunately, with this trust, also came the increase in phishing attacks using NHS logos, luring emails about vaccines and text messages stealing people’s personal information, etc. for fraud.

Looking to the future, we need to improve user resilience against phishing attacks without disrupting the trust aspects required amongst users for a thriving digital society. As Riegelsberger et al. [34, p.1] showed ‘there are cues in the user interface that can help to build trust to some extent (trustbuilders), and some cues that have a great potential for destroying trust (trustbusters)’. The authors of this paper feel that some of the answers need to lie in how we design and develop our online spaces. As a society, we need to push for a digital existence that enables users to effectively detect threats like the phishing attack and then to be able to trust and distrust accordingly. We need to harness the technology to support the end users whilst they exist online. It is not just about making these spaces usable, we need to think beyond usability and ensure that people are safe and secure. The authors feel that along with effective anti-phishing training, we need to push for usable security and online warnings that will support end users in making informed decisions about their own safety online.
